# Morphology prediction of small nanoparticles in any orientation from single electron micrographs

**DOI:** 10.1038/s41524-026-02114-w

**Published:** 2026-05-06

**Authors:** Henrik Eliasson, Fangjinhua Wang, Xi Wang, Daniel Barath, Marc Pollefeys, Rolf Erni

**Affiliations:** 1 Electron Microscopy Center, Empa – Swiss Federal Laboratories for Materials Science and Technology, Überlandstrasse 129, Dübendorf, Switzerland; 2 Computer Vision and Geometry Group, Department of Computer Science, ETH Zurich, Universitätstrasse 6, Zürich, Switzerland; 3 Machine Perception Research Laboratory, HUN-REN SZTAKI, Budapest, Hungary

**Keywords:** Materials science, Nanoscience and technology, Physics

## Abstract

Accurate and automated data analysis for transmission electron microscopy will enable new high-throughput experiments that can reveal atomic-scale structure–property relationships for many functional materials. A key challenge in this pursuit is scalable three-dimensional structure prediction from single two-dimensional images. Existing tomographic and atom-counting approaches require either high electron doses, complex acquisition schemes, or the object in specific orientations, limiting experimental design. Here, we introduce a diffusion-based generative workflow that predicts the three-dimensional morphology of nanoscale objects directly from a single scanning/transmission electron micrograph. Applied to sub-5 nm platinum nanoparticles on ceria, it successfully predicts reasonable structures across diverse particle morphologies and imaging orientations. Combined with automated data acquisition in *operando* experiments, we believe techniques like this could be an essential part in relating ensemble-level structural variation and dynamics with performance, particularly fitting for heterogeneous catalysis.

## Introduction

The rational design of functional materials requires a detailed understanding of atomic-scale structure–function relationships^1,2^. If such relationships could be established directly from experiment in a fast and simple way, it would provide the essential link between structural descriptors and performance needed to improve many systems through guided design, without the need for massive theoretical or experimental efforts. Using heterogeneous catalysts as an example, the experimental establishment of structure-function relations would require comprehensive and accurate characterization of the catalyst active sites paired with corresponding measurements of catalytic performance. This is in principle possible with operando transmission electron microscopy (TEM), but the high-throughput data acquisition and analysis workflows needed are not yet in place^3,4^. In supported metal catalysts, factors such as particle shape, size, dispersion, and metal–support interactions all strongly influence the properties of the material^5–7^. Consequently, a scalable technique that can provide accurate 3D descriptions of the supported species at the atomic scale in an automated manner is highly desired.

Scanning transmission electron microscopy (STEM) is among the most widely used and versatile techniques for obtaining direct structural information at the nanoscale. The high-angle annular dark-field STEM (HAADF-STEM) modality carries an easily interpreted mass-thickness contrast down to 50 pm in resolution, enabling direct imaging of atomic structures with inherent 3D information from the intensity-thickness relationship of the micrographs^8,9^. Quantitative 3D interpretation of single HAADF-STEM images is possible but has so far been mainly demonstrated for crystalline samples aligned along low-index zone axes^10–15^. For catalytically relevant nanoparticles below ~2 nm, however, it is rarely the case that particles are perfectly crystalline or conveniently oriented when encountered in the microscope. Electron tomography can, in principle, reconstruct 3D structure regardless of orientation, and has been successfully applied with atomic resolution to larger nanoparticles^16–30^. Yet, most small surface-supported nanoparticles are too beam-sensitive or structurally dynamic to survive the accumulated electron dose, and the acquisition time per particle remains prohibitive for high-throughput studies. This leaves a critical gap where we need a technique that is both agnostic to particle orientation and capable of predicting reasonable 3D structures directly from single STEM images.

Reconstructing a 3D object from a single view is a well-known problem in computer vision. The problem is ill-posed and relies on prior understanding to predict the occluded parts of the imaged object. Although the exact 3D structure of an object cannot be uniquely determined from a single view, recent generative approaches such as neural radiance fields and diffusion models have shown remarkable ability to produce high-quality, plausible reconstructions from images of everyday objects^31–33^. For example, PC^2^31^ introduced an image-conditioned point cloud diffusion model that, given a random gaussian point cloud and an image of an object of interest, learns to denoise the cloud of points into the shape of the object in the conditioning image, and assign color to the points accordingly. Representing objects as point clouds is not only significantly lighter computationally compared to dense 3D volumes, but it is also very fitting for atomic structures and molecules. Indeed, point-cloud diffusion models have recently been applied to problems like molecule conformation prediction^34,35^, and property-guided design of materials^36^. However, conditioning the diffusion process on images to recover the underlying structure that gave rise to the image, represents a different challenge. Such models have only recently begun to be explored in the context of microscopy for microscale objects^37,38^, while the atomic scale remains largely unexplored.

In this work, we utilize an image-conditioned point cloud diffusion model derived from PC^2 to predict the morphology of nanoparticles in HAADF-STEM micrographs. Platinum nanoparticles supported on CeO_2_ act as the model system and a large dataset of 256,000 images with paired atomic models is generated for training, 6,000 real multislice image simulations, and 250,000 synthetic multislice image simulations from a surrogate model^39–41^. The PC^2 framework is modified for STEM images and a center-of-mass free approach is employed for training stability^34^. The workflow is benchmarked on clean simulated data and synthetic experimental data created by passing the clean simulations through a style transfer model^4^, and finally the performance on real experimental micrographs is evaluated, showcasing the capabilities of the technique in generating 3D structures consistent with the conditioning HAADF-STEM image.

## Results and Discussion

### Micrograph-conditioned point cloud diffusion

Denoising diffusion models are trained to reverse a process in which noise is gradually added to clean data until only pure noise of a known distribution remains^42^. Once trained, a diffusion model can take random noise from that distribution as input and iteratively denoise it over a number of timesteps, *t*, to generate a new sample. Without conditioning, however, the generated samples will be random instances that fit into the training data distribution. By conditioning the diffusion process on information like text or an image, the model can be directed toward structures consistent with the conditioning^43^. In this work, our nanoparticle structures are gradually corrupted until the atom positions are essentially a gaussian distribution of points in space. When reversing the process, the diffusion model is conditioned on the HAADF-STEM image of the nanoparticle to guide denoising of the gaussian point cloud into a structure that fit with the image.

To find the underlying morphology of an imaged nanoparticle, we utilize a large dataset of 256,000 multislice image simulations with paired atomic models. All the atomic models are of platinum nanoparticles, randomly shaped and sized, in the 1-1000 atom range, supported on randomly shaped, oriented, and sliced CeO_2_ supports. The structure generation algorithm is explained in detail in previous work^4^. Out of the 256,000 samples, 6,000 are paired with real multislice image simulations, while the remaining 250,000 are paired with a synthetic multislice image generated by a surrogate model, also discussed in another work^41^, to speed up dataset generation. In this work, we are only concerned with the nanoparticles and not the supports, thus we select only the Pt atoms in the atomic models and use these as our ground truth structures. For morphology prediction, we choose to work with the Pt structures as point clouds rather than turning them into voxel volumes, to save computational resources. How to define morphology with a point cloud is a choice whether to sample points from e.g. the convex hull of the nanoparticle, or to use the raw particle structure directly. We choose to work with the raw particle structure as target because using the convex hull or variations of it will limit us to convex shapes or introduce new hyperparameters that need tuning. Working with the raw structures also keeps point density fairly consistent for each sample regardless of particle size, which we found helpful for model convergence. Furthermore, by using the atomic model as target, we in principle also define a workflow that is compatible with direct atomic structure prediction, which is a very interesting future direction, but not the goal of this paper and would likely require further advanced additions to the workflow.

### The diffusion model

For the diffusion model, a point-voxel CNN (PVCNN)^44^ was used (as in PC^2^31^) and the typical 1000 timesteps were used. For a full mathematical description of the ideas behind denoising diffusion probabilistic models and the optimization objectives, we refer to other works^31,41^. However, it is important to note that training and inference is done in different ways. During training, a random timestep *t* is selected for each example in the batch, and varying degrees of noise are added to each structure according to

1 $${{\bf{x}}}_{t}=\sqrt{{\bar{\alpha }}_{t}}{{\bf{x}}}_{0}-\sqrt{1-{\bar{\alpha }}_{t}}\epsilon ,\epsilon \sim N(0,I)$$

where **x**_0_ is the original atomic structure, **x**_t_ the noisy structure at timestep t and

2 $${\bar{\alpha }}_{t}={\prod }_{s=1}^{t}(1-{\beta }_{s})$$

is defined by the noise schedule $\{{\beta }_{t}\}$, which controls the variance of the Gaussian noise added at each timestep. As noise scheduler, we employ a custom schedule (see Figure S1) that puts more emphasis on smaller noise levels close to the ideal structure, giving the model more time to learn finer details of the structures. During training, the model is tasked with predicting the added noise ${\boldsymbol{\epsilon }}$ from the noisy input ${{\bf{x}}}_{t}$. During inference, structure generation is performed by iteratively denoising an initially Gaussian-distributed point cloud. At each timestep, the model predicts the noise present in the current structure, which is then used to update the point cloud toward a cleaner configuration. This process is repeated sequentially for all timesteps until $t=0$, yielding the final predicted atomic structure.

Catalyst samples never display perfectly homogenous nanoparticle size and shape distributions, so the workflow must deal with variably sized inputs. To handle nanoparticles with different numbers of constituent atoms, all clouds were padded with (0,0,0) points such that each contains 1024 points. This is necessary as PVCNN expects consistently sized inputs. To keep track of padded points during inference and training, a fourth binary dimension is added to the cloud where 0 denotes a padded atom and 1 denotes a real atom. Padded points are seen by the model but ignored in visualizations, loss calculations, and when removing the center of mass of the structure as discussed in the next section on conditioning. For sampling with experimental images, where the number of atoms of the nanoparticle is unknown, we utilize a nanoparticle size estimator that predicts the number of atoms from the image (model performance presented in Table S1), telling us how many points to assign “1” to^4,41^.

The diffusion model is followed by a second PVCNN refinement model which acts as a one-step refinement model, cleaning up the structures predicted by the diffusion model and outputting better structures. To train the refinement model, ground truth structures were corrupted with noise designed to mimic the shortcomings observed in the first diffusion model output. As a final step, we minimize the variance of nearest neighbor distances in the predicted structure while keeping the xy positions locked. This is done to avoid having points unphysically close to each other and promote a consistent nearest neighbor distance, as expected for FCC platinum.

### Conditioning

We employ a similar approach to conditioning as in PC^2^31^ and GECCO^32^ and utilize a convolutional neural network (CNN) to extract multi-scale features from the conditioning STEM image. We are interested in reconstructing a 3D nanoparticle morphology, and we want the feature extractor model to extract useful features for that task. Therefore, we use a U-Net and train it to predict per-pixel thickness of the nanoparticle in the image (representative performance visualized in Fig S2). This task not only teaches the model to mask out the particle region, but also to predict the thickness of the nanoparticle in each pixel, which contains valuable 3D information. To condition the point cloud on the image and feature maps, all feature maps from the encoder side of the U-Net are upscaled to the input resolution and form a stack of images. The final particle thickness map is also added to this stack of images. The point cloud is projected onto the image-feature map stack before each diffusion step to sample local feature values from them. The points in the cloud have the pixel values from the pixel in the stack onto which they were projected, concatenated to them as features. Points projected onto different pixels of the image will thus have different features, carrying relevant information about that specific location in the image. We found that utilizing all feature maps was redundant and used only the ones from the 1st and 3rd encoder step. After concatenating features to the points, the point clouds have 325 features, 3 from the points’ cartesian coordinates, 1 to discriminate real from padded points, 320 features from feature maps at the 1st (64 channels) and 3rd (256 channels) encoder steps of the thickness prediction model, and finally 1 from the actual pixel value of the predicted thickness map.

**Fig. 1:**
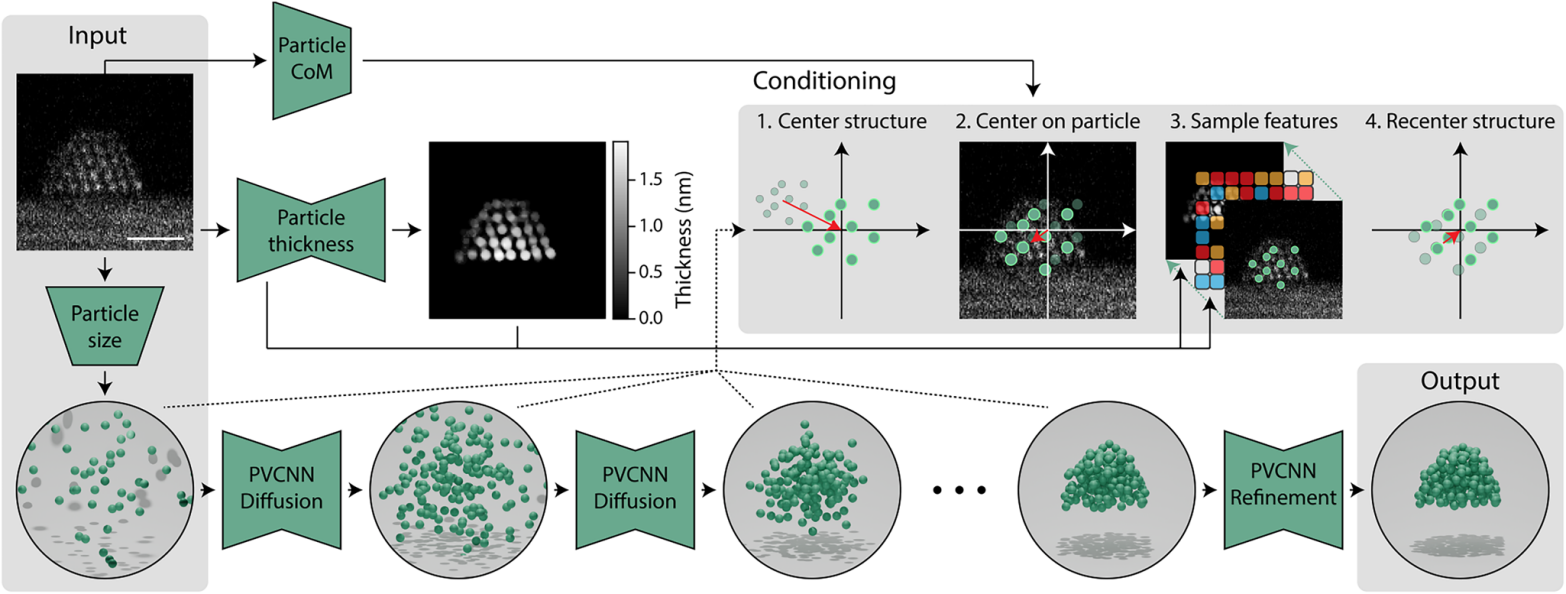
A schematic visualization of the workflow and how sampling works given a gaussian point cloud input along with a conditioning image. First, three networks extract necessary information from the conditioning image, the center of mass of the particle in the image, a thickness map, and a predicted size of the particle in number of atoms n_atoms_ (183 in this example). The input point cloud consists of 1024 points generated from a 3D gaussian distribution and have 1 assigned to the 4^th^ channel of n_atoms_ of the points while the rest are assigned 0. The center of mass of the non-padded points is subtracted from all points and the structure point cloud is then shifted in xy such that the center of mass of the cloud is geometrically located on top of the predicted center of mass of the particle in the image. The cloud is conditioned by sampling features from feature maps and output of the thickness predictor model, and the structure is then shifted back to 0 CoM. The cloud is then passed into the diffusion model which predicts the noise we need to remove from the noisy point cloud. This is repeated for a number of timesteps and then followed by a final one-step refinement model, giving us the final output. The scale bar represents 1 nm and the whole denoising for this example can be seen in Video S1.

The nanoparticle can be located anywhere in the conditioning image frame, which means that the diffusion model must learn to find where in xy the particle is, to bring the points there during denoising. This is an unnecessary challenge for the model and both speed of convergence and quality of the predictions were improved by adopting a center-of-mass-free workflow so that features are more consistently sampled from the particle region of the image. To realize this, a U-Net was trained to find the center of mass (CoM) of the nanoparticle in the conditioning image (representative performance visualized in Figure S3), and this CoM was used to offset the point clouds such that the xy-mean of the point clouds always aligned with the xy of the CoM in image coordinates before sampling features. After sampling, the point clouds are shifted back to 0 mean before being passed into the diffusion model. The entire conditioning, diffusion, and refinement scheme is schematically visualized in Fig. 1.

### Evaluating structure prediction performance on synthetic data

From the initial dataset of 256,000, five percent were used for validation (mix of real and synthetic multislice images) and 1,000 examples from the real multislice set were set aside for testing and evaluation. To ensure that the trained models generalize to experimental data, all images were passed through a cycle-consistent generative adversarial network (CycleGAN) trained to map images between the noise-free style of raw multislice images and the specific noise profile of the experimental data^4,43,45^. Passing the clean simulated images through the CycleGAN yields synthetic experimental images that look realistic and have consistently scaled pixel intensities. Both image styles are visualized in Figure S4.

**Fig. 2:**
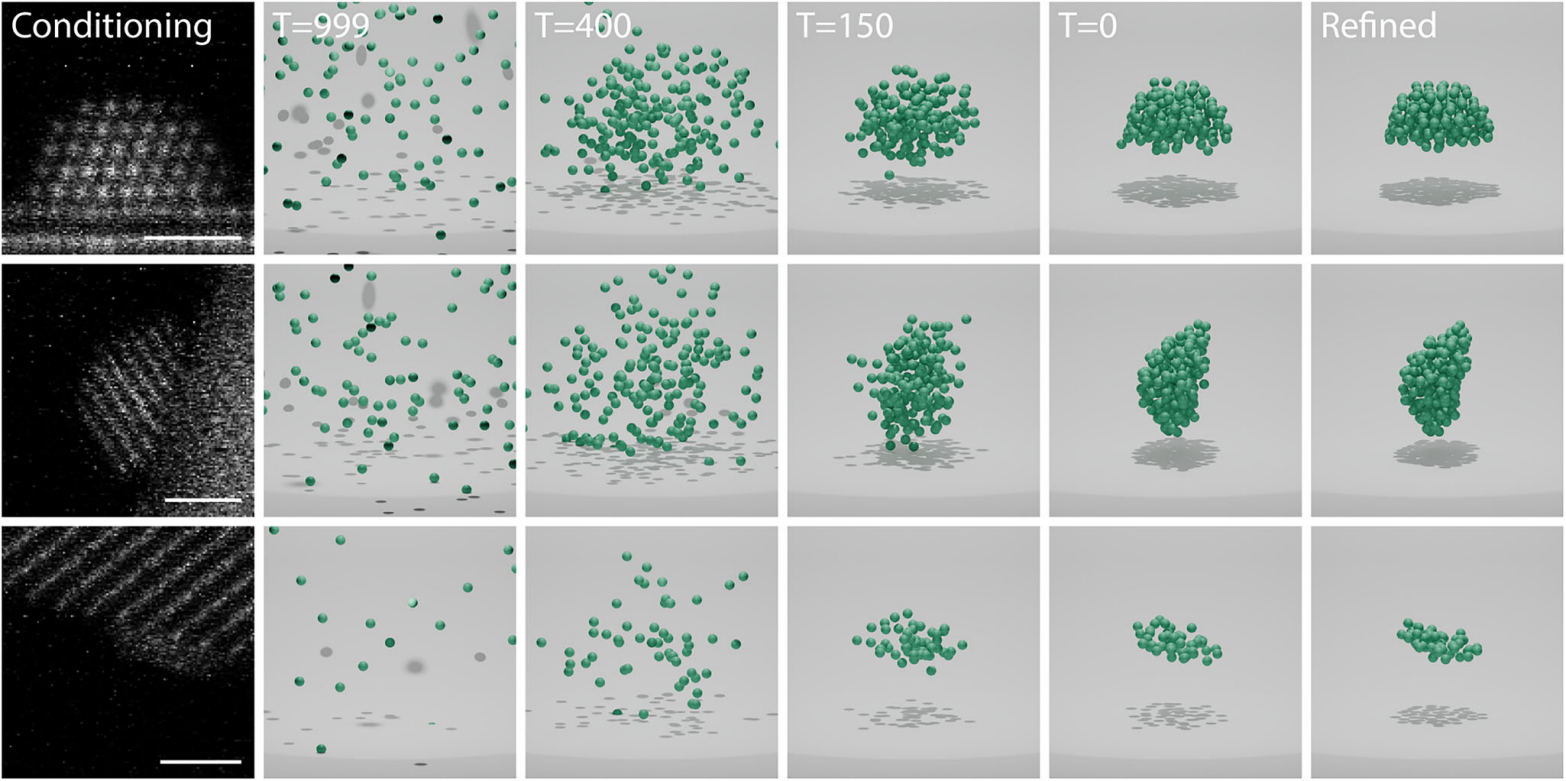
Intermediate steps in the diffusion workflow for three synthetic experimental HAADF-STEM images. Scale bars represent 1 nm. As ground truth structures are available when running the workflow with synthetic experimental data, the performance can be further evaluated. Qualitatively, Fig. 3 displays morphology predictions along with ground truth for five examples. In general, predictions fit the conditioning very well in the image plane and the 3D shape is typically reasonable. The more lattice information that is available in the conditioning image, the more accurate the generated structures are, and certain particles imaged in zone axis are almost perfectly reconstructed (e.g. Fig. 3a). Morphology is largely correct regardless of how much crystal information is available in the image, both in xy and along the beam direction. The shape of the prediction in the image plane matches well with the particle in almost all cases and the shape is generally more accurate in xy than along z, which is to be expected as there is no conditioning available to help the model along that direction.

The dataset consists solely of Pt nanoparticles supported on cerium dioxide. These particles range from 1 to 1000 atoms in size and are oriented in any orientation with respect to the electron beam. All particles are imaged in profile over vacuum, and the particle-support interfaces have small misstilts of up to 10° w. r.t the electron beam. All structures are based on FCC Pt and fluorite CeO₂, with varying degrees of applied perturbations, resulting in some structures being perfectly crystalline while others are more disordered and some with a complete lack of short-range order. A key strength of the proposed technique is that it can handle particles imaged in any orientation and in a range of sizes. Figure 2 displays how the model iteratively denoises a gaussian point distribution into reasonable morphologies consistent with the conditioning image for three particles, 205, 195, and 52 atoms in size, imaged in different orientations. The first particle is oriented to a major zone axis and displays the typical atomic columns with increased pixel intensities due to channeling contrast, the second particle is imaged in a non-trivial orientation, however, still displaying lattice planes. The third structure is very small, amorphous-looking and non-trivial to analyze, even for a domain expert. Our workflow succeeds in generating a plausible morphology for all cases.

**Fig. 3:**
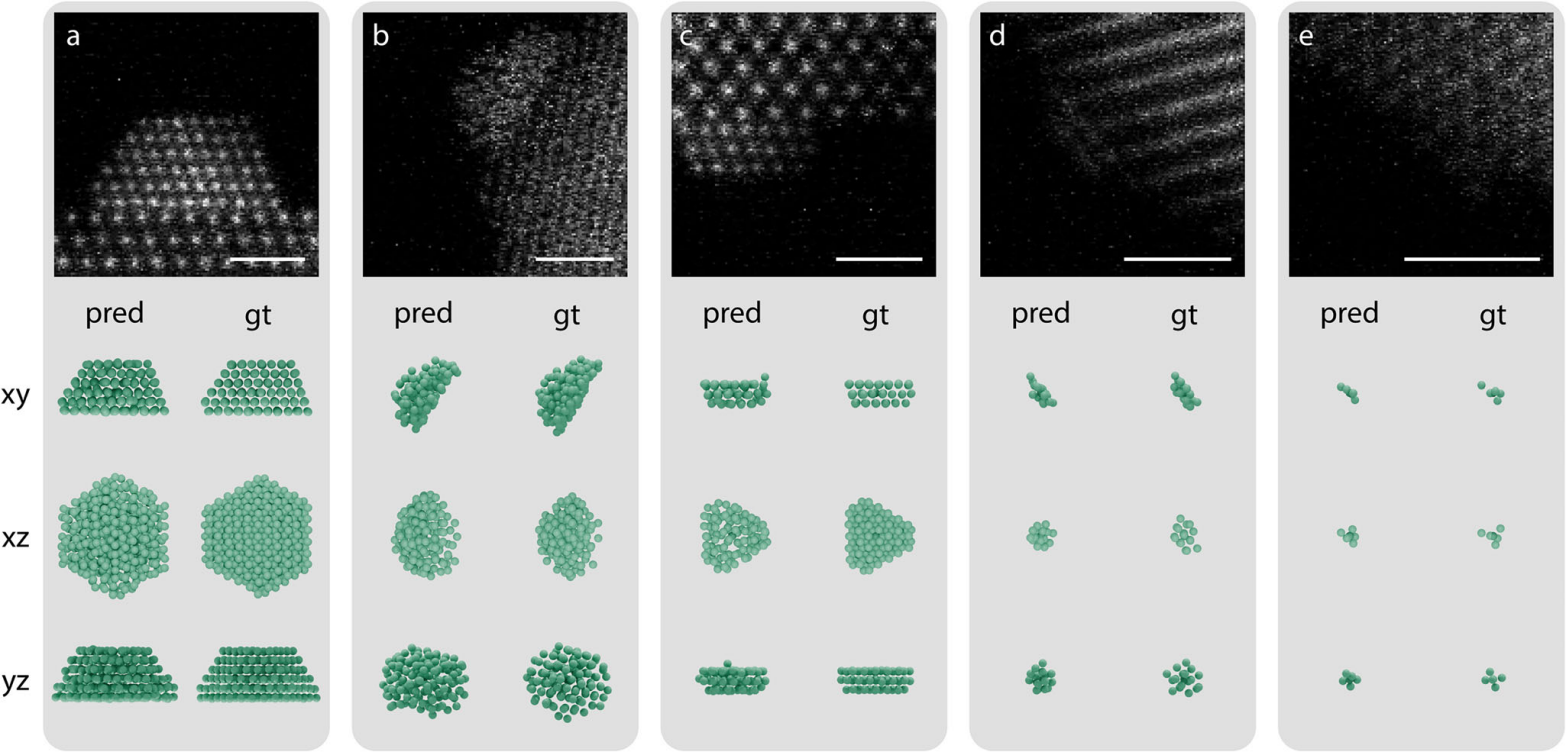
Comparison between predicted structures and ground truth structures along three different views for five examples. **a** 401, **b** 140, **c** 94, **d** 17, **e** 5 atoms in size. To quantitatively benchmark the workflow, we use the chamfer distance (CD) between predicted and ground truth structures in 3D, and in 2D after all atoms have been projected onto the image plane (CD_2D_). A better CD indicates that the predicted point cloud better matches the ground truth atomic structure and a better CD_2D_ indicates that the prediction better fits the conditioning image. As a baseline to compare with, the predicted thickness map is used to define a symmetric 3D shape by placing points in space at positive and negative half of the thickness at each pixel. These points make up a convex hull in which we sample point positions to form the baseline point cloud. Example baseline structures along with predictions and ground truths can be seen in Figures S5-S10. Mean chamfer distances for the workflow trained with different beta schedulers, with clean and synthetic experimental image data, and using different numbers of timesteps for sampling were calculated on the test set of 1000 examples and is presented in Table 1.

We find that the diffusion model consistently outperforms the random baseline by a large margin in both 3D and 2D Chamfer distance. The best-performing configuration is obtained using the custom β-schedule and only 100 inference timesteps, yielding a CD of 0.0350 and a CD_2D_ of 0.0046. Interestingly, using 100 timesteps as opposed to 1,000 that the model was trained with, yields slightly better and more robust results. This is a benefit as it essentially makes inference 10x faster. The reason that fewer timesteps is better for this custom noise schedule is likely due to propagating errors, as many timesteps close to T = 0 have extremely similar noise levels. In contrast, when using a linear β-schedule where using all 1000 sampling steps yields the best results. When trained and evaluated on clean data, the model achieves the lowest overall error (CD = 0.0320, CD_2D_ = 0.0031). We see these results as a lower bound for the performance of the workflow in its current form, representing the best achievable prediction accuracy in the absence of noise.

**Table. 1:** Model performance on clean data and synthetic experimental data for different number of inference timesteps, beta-schedules, and conditioning image style

Image type	β-Schedule	Timsteps for sampling	CD	CD_2D_
Clean	Custom	100	0.0320	0.0031
Noisy	Linear	100	0.0371	0.0058
Noisy	Linear	1000	0.0355	0.0048
Noisy	Custom	10	0.0426	0.0075
Noisy	Custom	100	**0.0350**	**0.0046**
Noisy	Custom	1000	0.0363	0.0049
Noisy	—	—	0.0475^a^	0.0095^a^

^a^Baseline.

Since the diffusion model is probabilistic in nature, it is also of interest to investigate the variation among different predictions generated from the same conditioning image. Fig S11 shows five independently sampled predictions for a representative example, illustrating the model’s probabilistic but consistent behavior. To further visualize the structural variability, Fig S12 displays an overlay of 25 predicted structures, revealing that while predicted atomic coordinates exhibit positional spread between samples, the overall morphology is highly consistent and realistic.

### Applying the workflow to experimental data

**Fig. 4:**
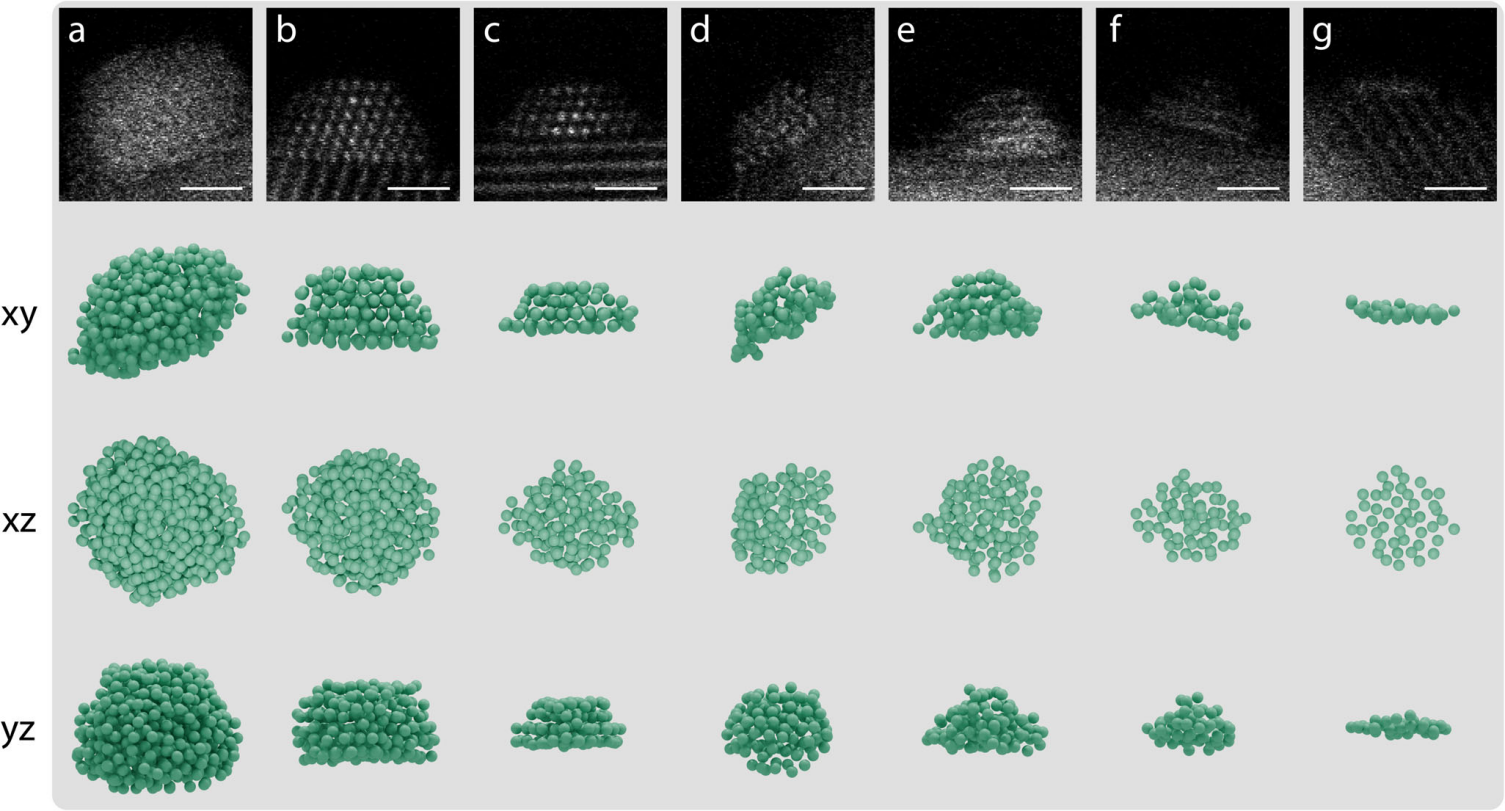
Predicted structures for 7 different experimental images. The structures are predicted to be **a** 624, **b** 331, **c** 144, **d** 132, **e** 122, **f** 66, and **g** 46 atoms large. Scale bars represent 1 nm.

The synthetic experimental data used to train the diffusion workflow was created using a CycleGAN trained on a specific experimental noise profile. This ensures that the workflow generalizes directly to real experimental data acquired with those acquisition and instrument parameters. Predictions for 7 experimentally observed structures are visualized in Fig. 4. From these predictions it is evident that the workflow indeed generalizes well to experimental data and a wide variety of particle shapes, sizes, and imaging orientations can be handled. Again, running the model several times with the same conditioning image also reveals a consistent morphology, shown in Fig S13.

**Fig. 5:**
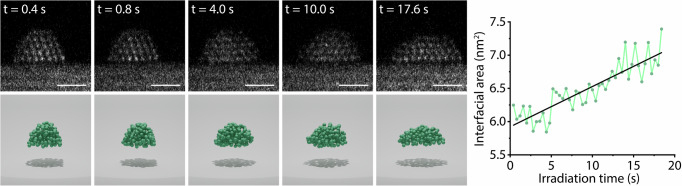
The diffusion workflow applied to selected frames of an experimental time-series of a Pt particle on a CeO_2_ support. The particle is estimated to be 183 atoms large and the scale bars represent 1 nm. Tracking the interfacial area between the nanoparticle and the support reveals that, after a short initial stable period, it increases steadily under electron irradiation at a rate of approximately 0.06 nm²/s.

The workflow enables applications like tracking shape over time, providing insights into electron irradiation effects, fluxional behavior, and potential reactions to other stimuli like gas and temperature. Since our technique works on single images, unrivaled temporal resolution can be achieved. Each frame from a time-series of a single Pt nanoparticle recorded at 2.5 frames per second was analyzed by the workflow, and five selected frames are displayed in Fig. 5. The entire sequence is played back in real time in Video S2. As an example, we analyze how the interfacial area between catalyst nanoparticle and support changes under electron irradiation.

To generate structures for each frame, we assume there is no loss of atoms throughout the series and use the mean predicted particle size and the same initial noise distribution to create the input point clouds to the diffusion model. The dose rate in this experiment, using a beam current of 35 pA, dwell time of 1 µs, and a pixel size of 25 pm, was 3.5 × 10^9 ^e/Å^-2^/s^-1^. The particle maintains its structure for the first seconds after which the interfacial area steadily increases at a rate of 0.06 nm^2^/s. At the same time, the images show how the structure indeed spreads out and goes from 5 atomic layers to 4 in height. This evolution is most likely driven by electron irradiation, and the initial stability of the structure is interesting. It is hinting at either local heating or charge accumulation eventually becoming large enough to prompt the reconstruction into a structure with a larger particle-support interface. Electron beam effects on this scale are poorly understood in STEM, and decoupling effects of the beam from those of temperature and reactive atmospheres is crucial for fundamental studies in catalysis. With workflows like the one presented herein we are one step closer to bridging theoretical and experimental studies to bring answer to these questions.

### Practical considerations

The presented workflow is trained specifically for platinum nanoparticles. This means that to use the workflow for any other system, new training data must be generated for that specific system, and all models be retrained. Generating training data is a bottleneck if computational resources are limited, and generating 100,000 new images could take anywhere from an hour to weeks depending on image size, hardware acceleration, and number of parallel simulations. All models presented in this work were trained on a single RTX4090 GPU, where going from random noise to predicted structure took on average 6 s. Of these 6 s, the 100 passes through the model stands for the majority of the computation time and is where the most time can be saved. With smarter inference and distillation tricks like in modern diffusion based image generators^46^, the number of passes needed could likely be reduced below 10 in the future, reaching sub-second prediction speed.

The workflow can also be adapted for custom particle size ranges other than 1–1000. We set the size limit of the point clouds to 1024. That is not a hard limit and can be set higher at increased computational cost. However, with a large range of partice sizes, there is also a spread in size estimation performance as seen in Table S1. For analysis of only larger particles, or only smaller particles it would probably be beneficial to tune the distribution of sizes in the training set to around what is expected for the experimental data. For example, to use the workflow for a low-nuclearity or single atom catalyst, one could train the workflow solely on clusters in the 1–10 atom range rather than up to 1000 as we have.

Finaly, there are limitations to what information is present in a single HAADF image, especially in 3D along the beam direction. It is known that the HAADF signal carries mass-thickness information. Under the assumption that the particle consists of a single element, it should be possible to infer thickness with high accuracy. Because of this, the conditioning image informs the model strongly how the structure should look in xy, but also provides a weaker conditioning along z, as it can deduce the thickness of the structure from the image. This seems sufficient for morphology prediction, and our model can handle particle structures that are not mirrored in the image plane fairly well. Why it does not always produce structures that are mirrored in the image plane is likely due to biases picked up from the dataset, or fine details in the image that give away information of which atoms are in front of others.

One should think of the structure predictions as probabilistic and not deterministic. Where there is conditioning, the model needs to hallucinate less to make up a structure. An important question for the future is how to further adapt the workflow to make the predicted structures reliable. The two most promising ways of grounding the model are in our opinion (1) to ensure that the predicted atomic structure gives rise to the conditioning image when it is passed through an image simulator, and (2) include physical potentials to encourage the formation of lower energy configurations. The first could likely be realized with a differentiable surrogate image generator, but how to define a well-behaving loss function for intermediate diffusion steps is not straightforward.

## Methods

### Machine learning

The diffusion model was trained with an *L*_2_ loss function between predicted and actual added noise ε, and the refinement model was trained using a Sinkhorn loss^47^ between ground truth structure and clean structure after subtracting the predicted final noise from the input structure. Both were trained with the AdamW optimizer and a cosine learning rate scheduler with an initial learning rate of 2e-5 for 100 epochs, and a batch size of 16. All models, including size predictor, thickness predictor, and CoM predictor, were trained and evaluated on a workstation equipped with a single NVIDIA RTX4090 GPU.

### Scanning transmission electron microscopy and image simulations

Experimental STEM images were recorded on a probe-corrected Titan Themis S/TEM microscope operated at 300 kV. The following acquisition parameters were used: a frame size of 512 × 512 and pixel sizes between 18.51 pm, a beam current between 35 and 40 pA, a convergence angle of 18.5 mrad, a beam dwell time of 1 µs, detector collection angle, gain, and offset of 72–200 mrad, 43.16 dB, and -1.8, respectively. The images were then cropped to 128 × 128 around the region of interest containing the particle.

Atomic structures of supported nanoparticles were generated by a custom algorithm explained in detail in another work^4^. Multislice images of size 128 × 128 were generated of the structures with pixel sizes ranging from 5 to 35 pm. The slice thickness was 0.1 nm, and 10 frozen lattice configurations were used.

## Supplementary information

Supporting Information.

Supporting_Video_1.

Supporting_Video_1.

## Data Availability

Data and model weights are available through Hugging Face at https://huggingface.co/datasets/Stemson-AI/Morphology_Prediction.

## Code availability

Code is available on GitHub (https://github.com/benke97/STEM2M).
