# Supplementary material for: Morphology prediction of small nanoparticles in any orientation from single electron micrographs
Source: NPJ Comput Mater. 2026 May 6;12(1):234. doi: 10.1038/s41524-026-02114-w (PMC13357205; doi:10.1038/s41524-026-02114-w)
Supplement: Supplementary file 1 — Supporting Information. [file 41524_2026_2114_MOESM1_ESM.docx]

# Supporting Information

**Morphology Prediction of Small Nanoparticles in any**

**Orientation from Single Electron Micrographs**

Henrik Eliasson^1*^, Fangjinhua Wang^2^, Xi Wang^2^, Daniel Barath^2,3^, Marc Pollefeys^2^ and Rolf Erni^1^

*^1^Electron Microscopy Center, Empa – Swiss Federal Laboratories for Materials Science and Technology,
Überlandstrasse 129, CH-8600 Dübendorf, Switzerland.*

*^2^Computer Vision and Geometry Group, Department of Computer Science, ETH Zurich,
Universitätstrasse 6, CH-8092 Zürich, Switzerland.*

*^3^Machine Perception Research Laboratory, HUN-REN SZTAKI, Budapest, Hungary*

**Table S1:** Median absolute percentage error (MAPE) of the nanoparticle size estimation predictions with different training datasets and for different particle size ranges. The training data contains particles between 1-1000 atoms and the metric is calculated for the subset of particles in the ranges 1-10 atoms, 10-100 atoms, and 100-1000 atoms.

| Image type | Number of Training images | MAPE  1-10  atoms | MAPE  10-100  atoms | MAPE  100-1000  atoms |
| --- | --- | --- | --- | --- |
| Clean | 255,000 | 19% | 5% | 3% |
| Noisy | 5,000 | 110% | 26% | 10% |
| Noisy | 105,000 | 60% | 15% | 7% |
| Noisy | 255,000 | 31% | 8% | 4% |


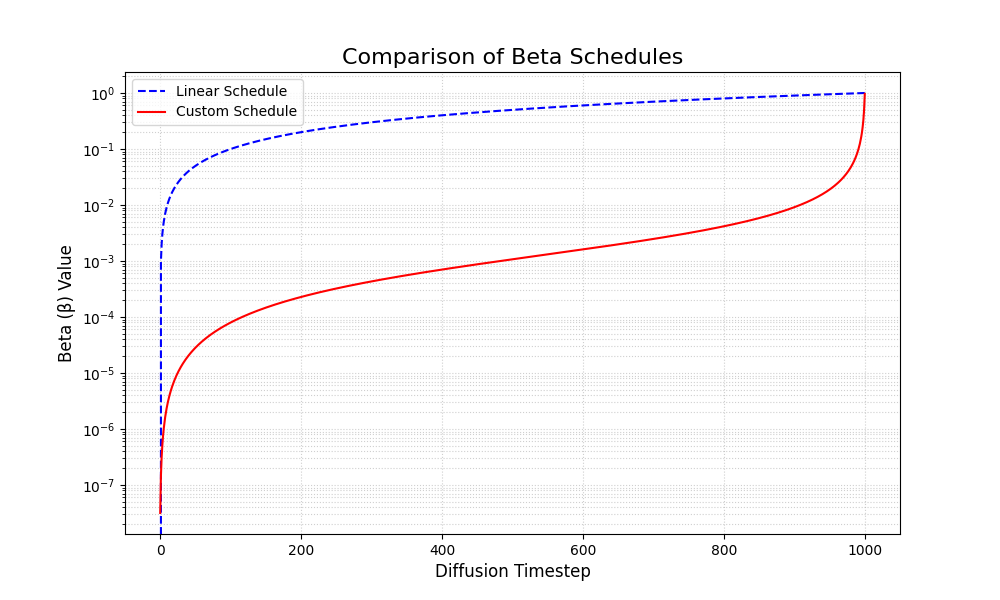


**Figure S1:** The custom beta scheduler used to train the diffusion model compared to a typical linear one. This custom schedule reduces the noise level much faster as the diffusion timestep T goes from 1000 to 0, letting the model see and train more on structures that are low noise and still retain crystalline information.


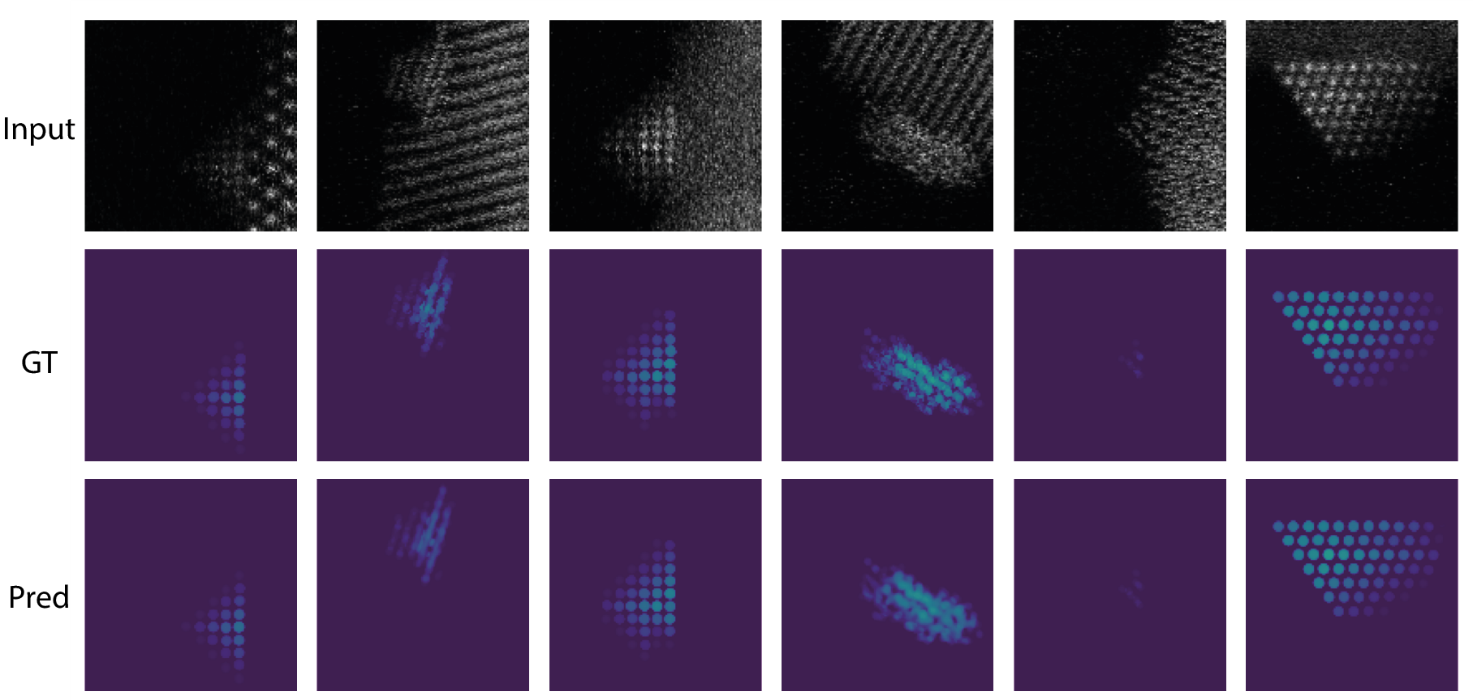
**Figure S2:** Representative performance of the particle thickness predictor. Given the input image in the top row, the trained network outputs the predicted thickness map in the bottom row. The middle row displays the ground truth.


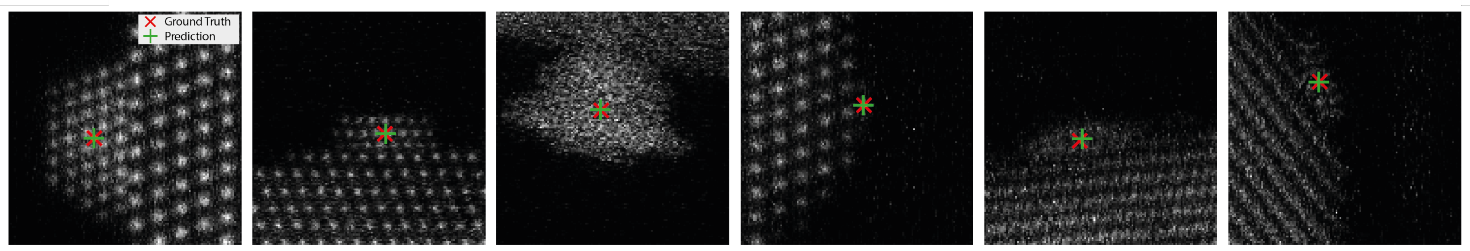


**Figure S3:** Representative performance of the center of mass predictor network. The red cross marks the ground truth and the green plus marks the trained network's prediction.


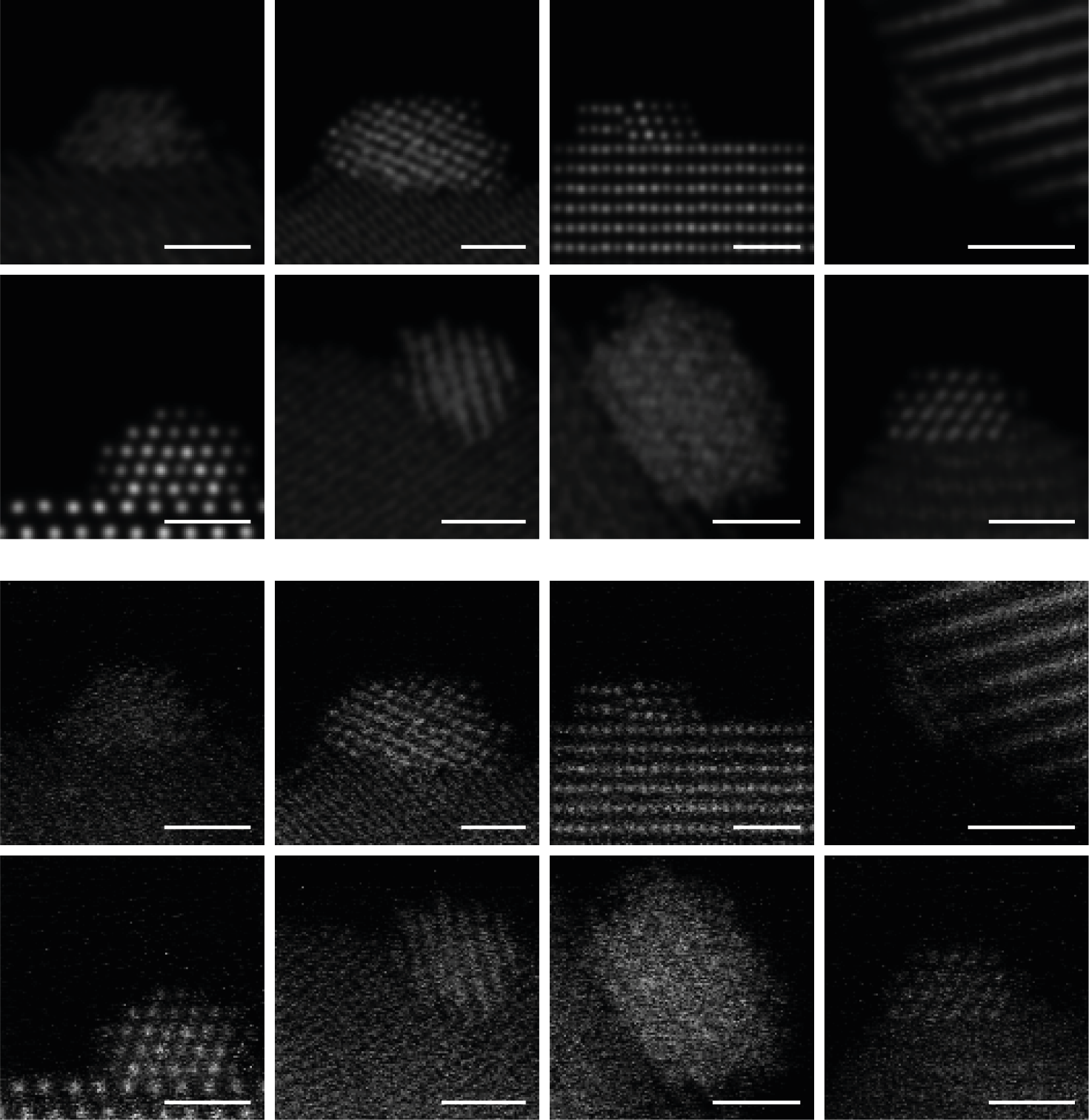


**Figure S4:** Eight example conditioning images from the test dataset in simulated and synthetic experimental styles. The top two row show the simulated images and the bottom row displays the corresponding images after mapped to the experimental domain by a cycleGAN.


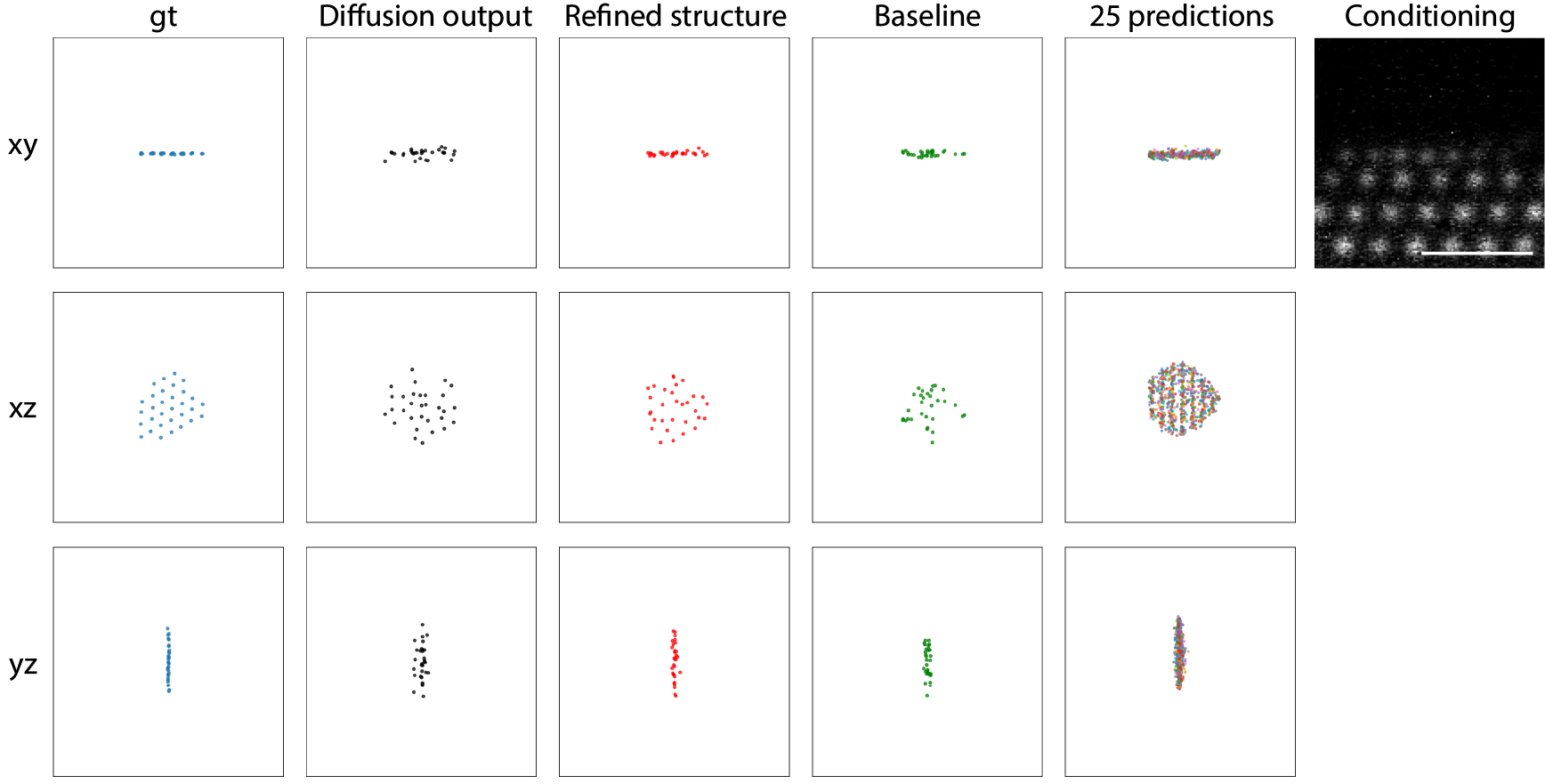


**Figure S5:** Comparison between the ground truth structure, the diffusion output, the refined structure, the baseline structure, and 25 refined structure predictions overlaid


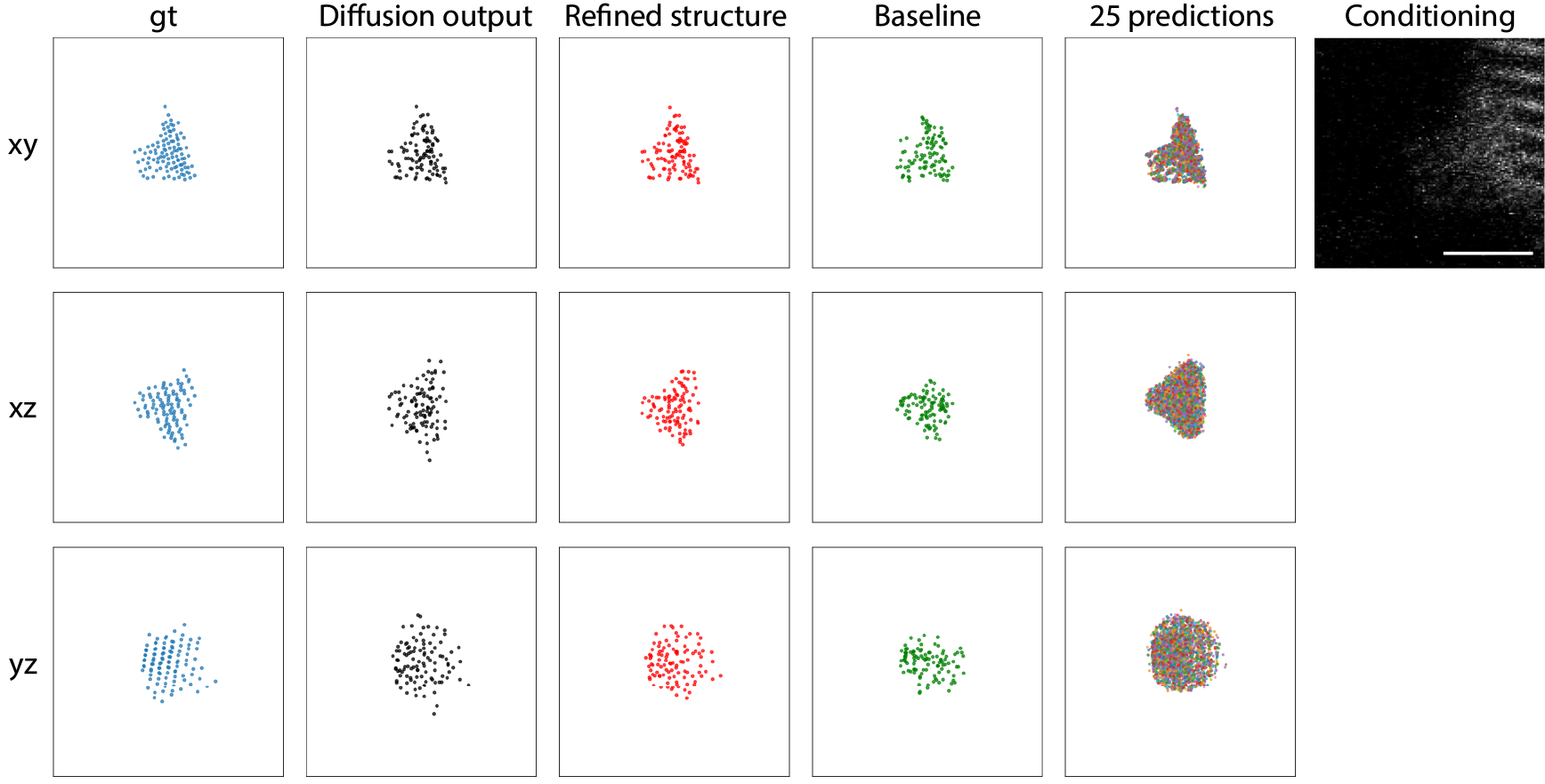
**Figure S6:** Comparison between the ground truth structure, the diffusion output, the refined structure, the baseline structure, and 25 refined structure predictions overlaid


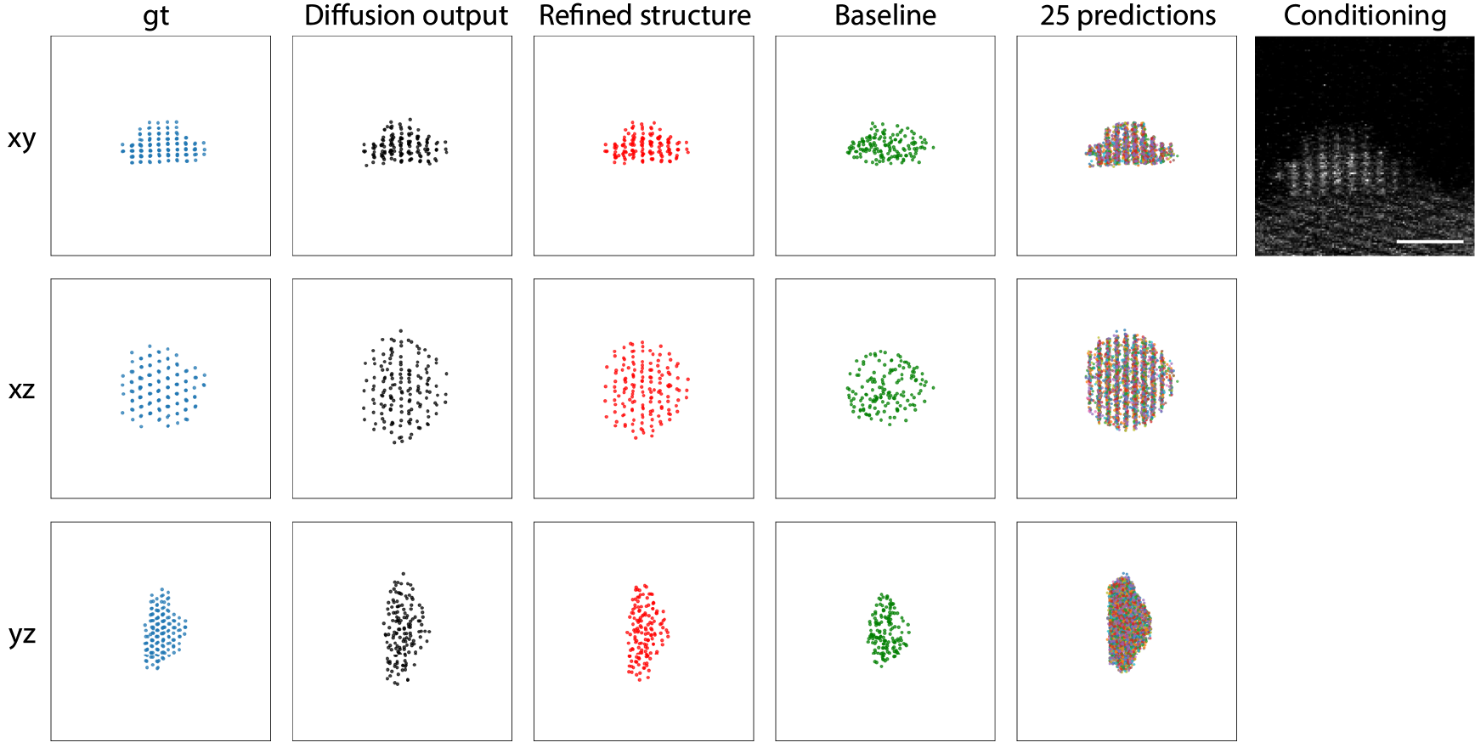


**Figure S7:** Comparison between the ground truth structure, the diffusion output, the refined structure, the baseline structure, and 25 refined structure predictions overlaid


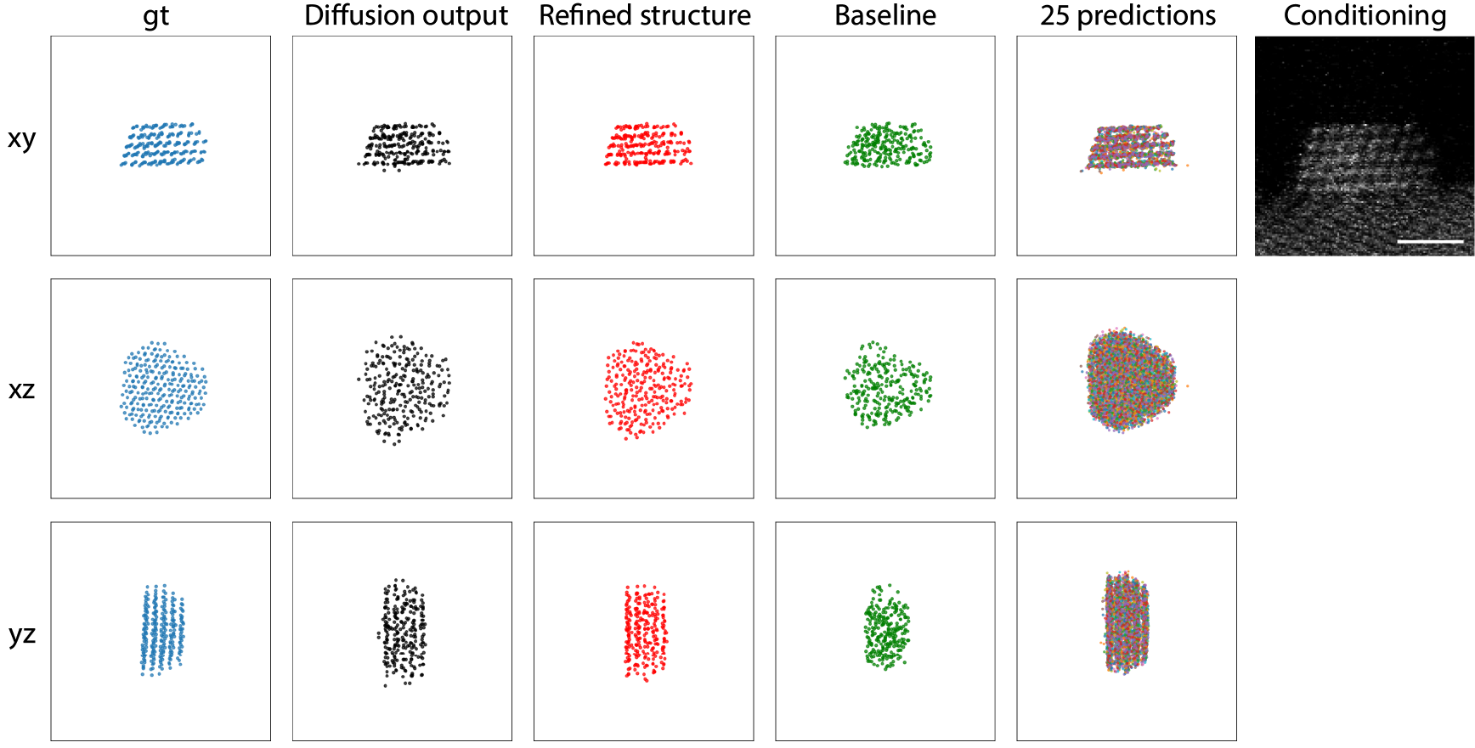


**Figure S8:** Comparison between the ground truth structure, the diffusion output, the refined structure, the baseline structure, and 25 refined structure predictions overlaid


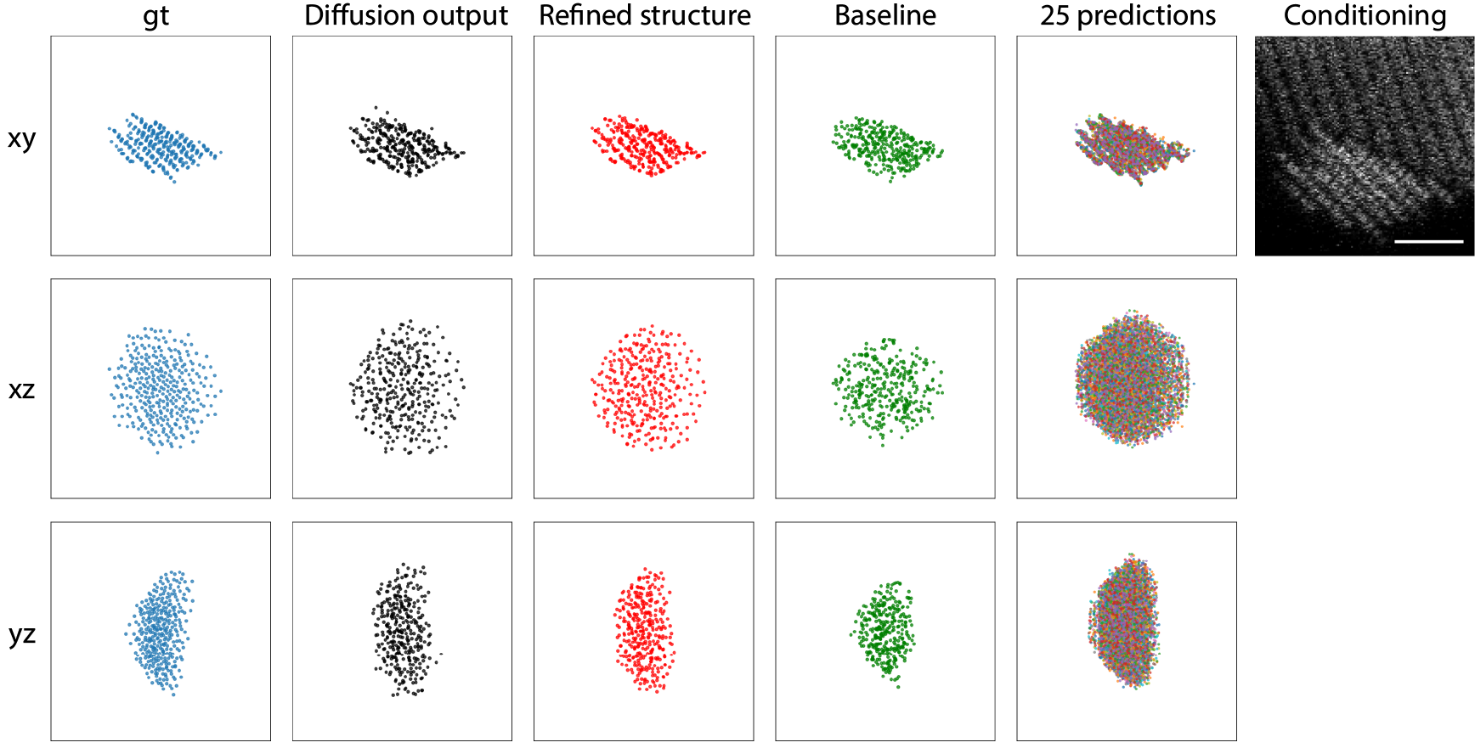
 **Figure S9:** Comparison between the ground truth structure, the diffusion output, the refined structure, the baseline structure, and 25 refined structure predictions overlaid


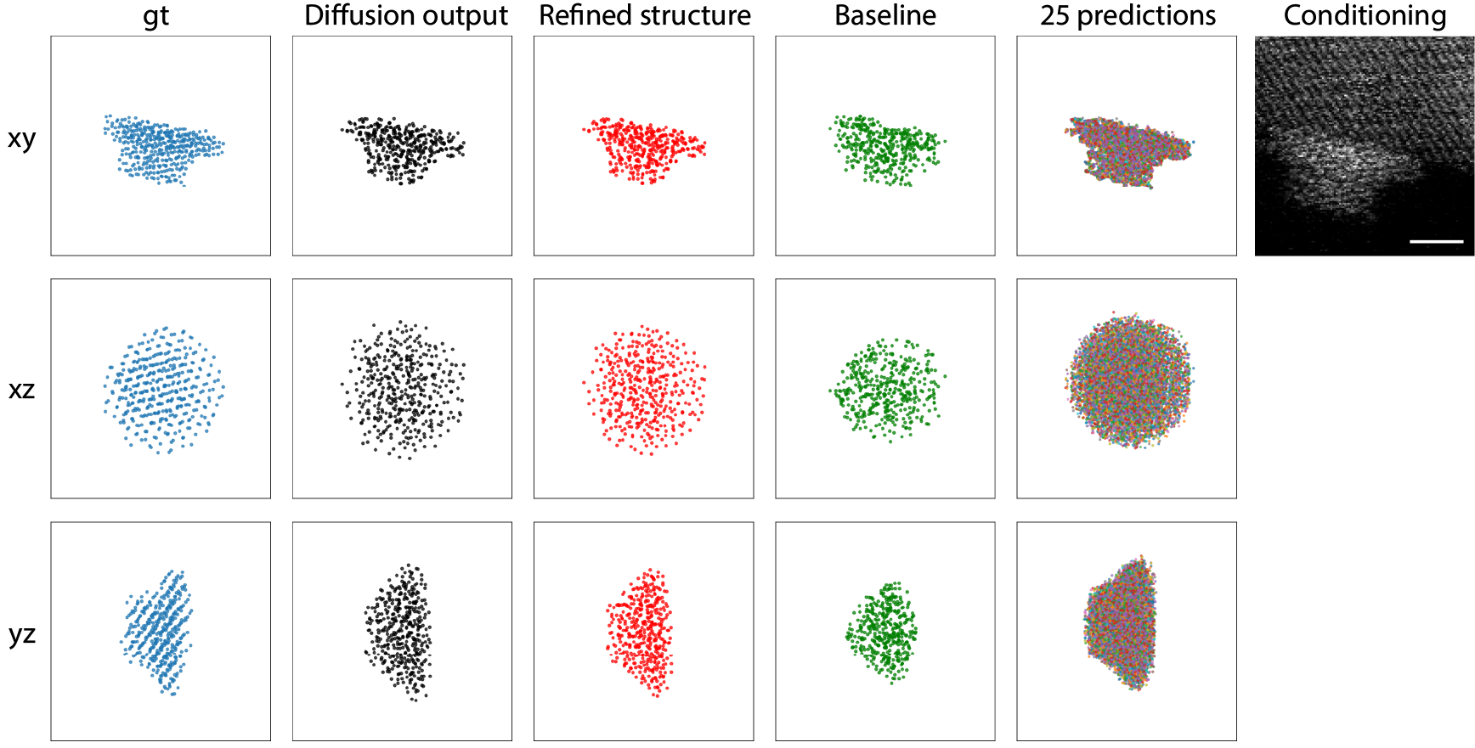


**Figure S10:** Comparison between the ground truth structure, the diffusion output, the refined structure, the baseline structure, and 25 refined structure predictions overlaid


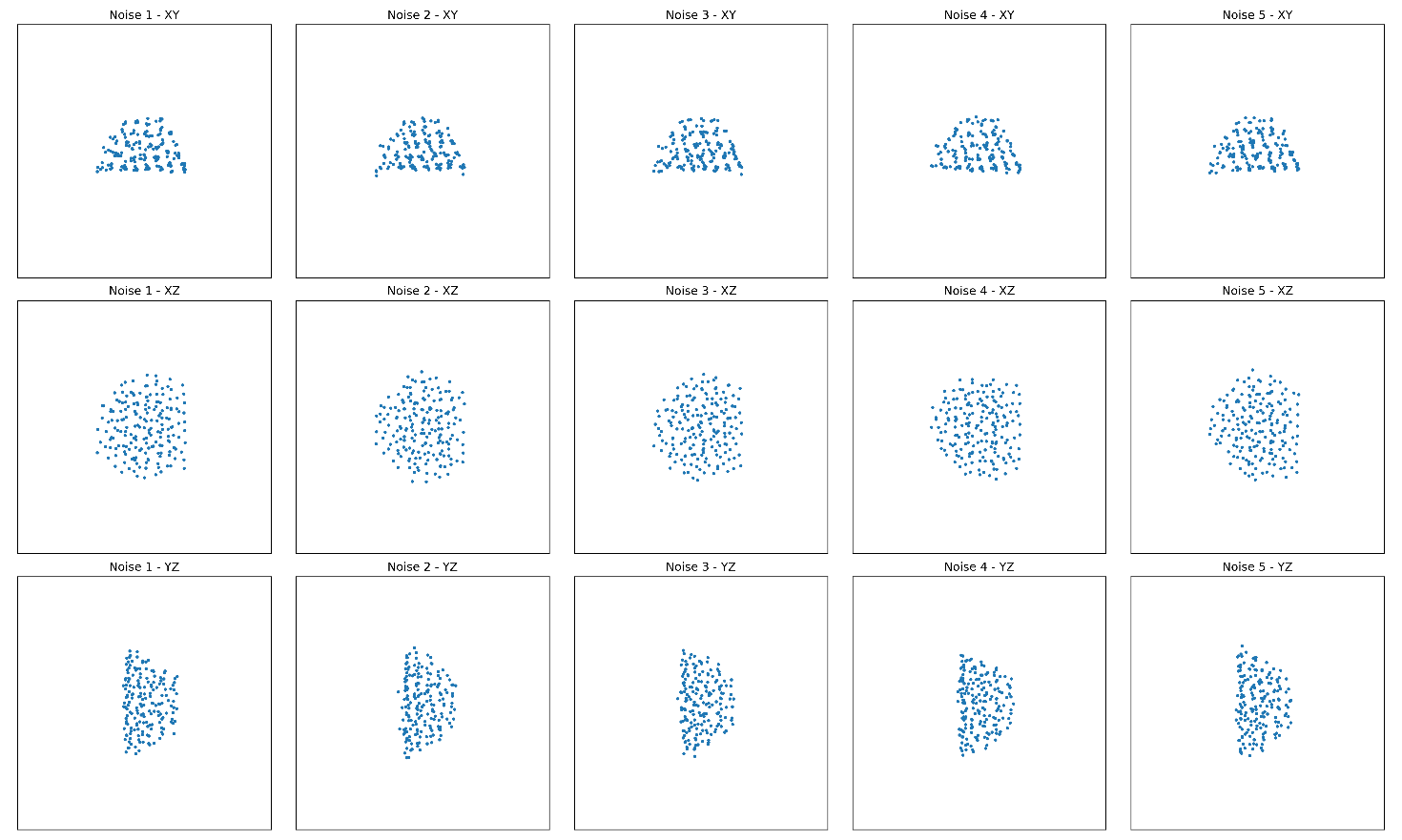


**Figure S11:** Five predictions given the same conditioning image but with different initial gaussian noise distributions.


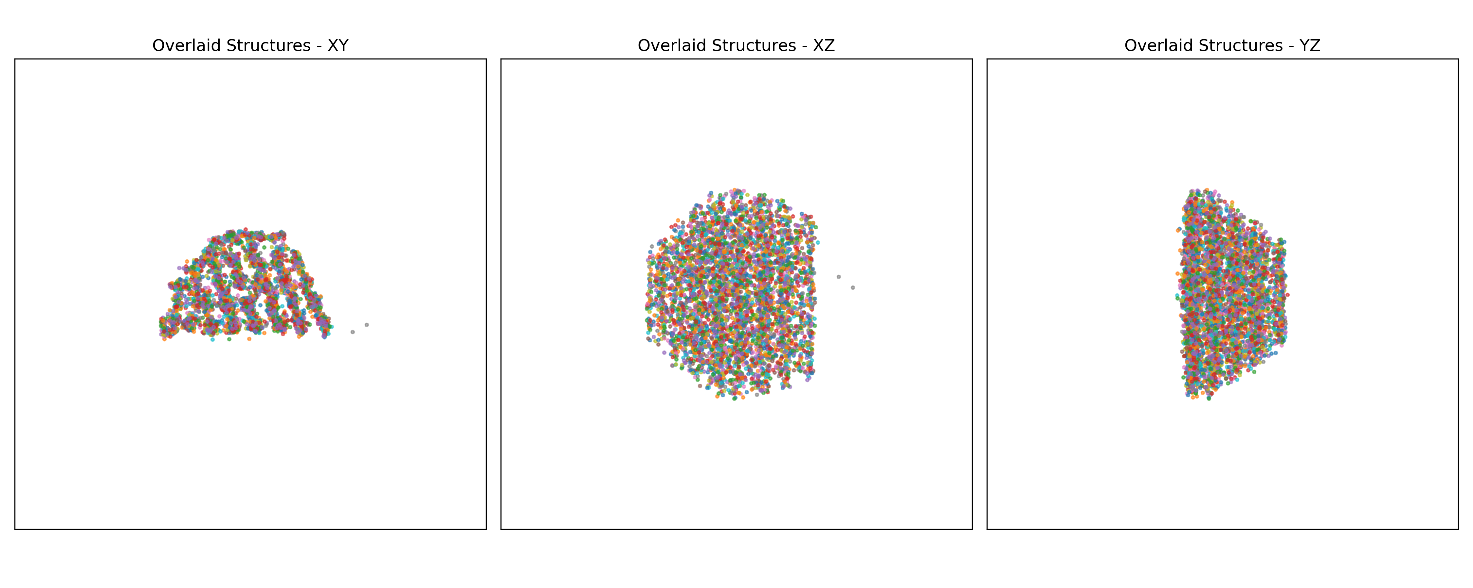
**Figure S12:** 25 structure predictions overlaid for the experimentally observed structure shown in figure 1 and 5.


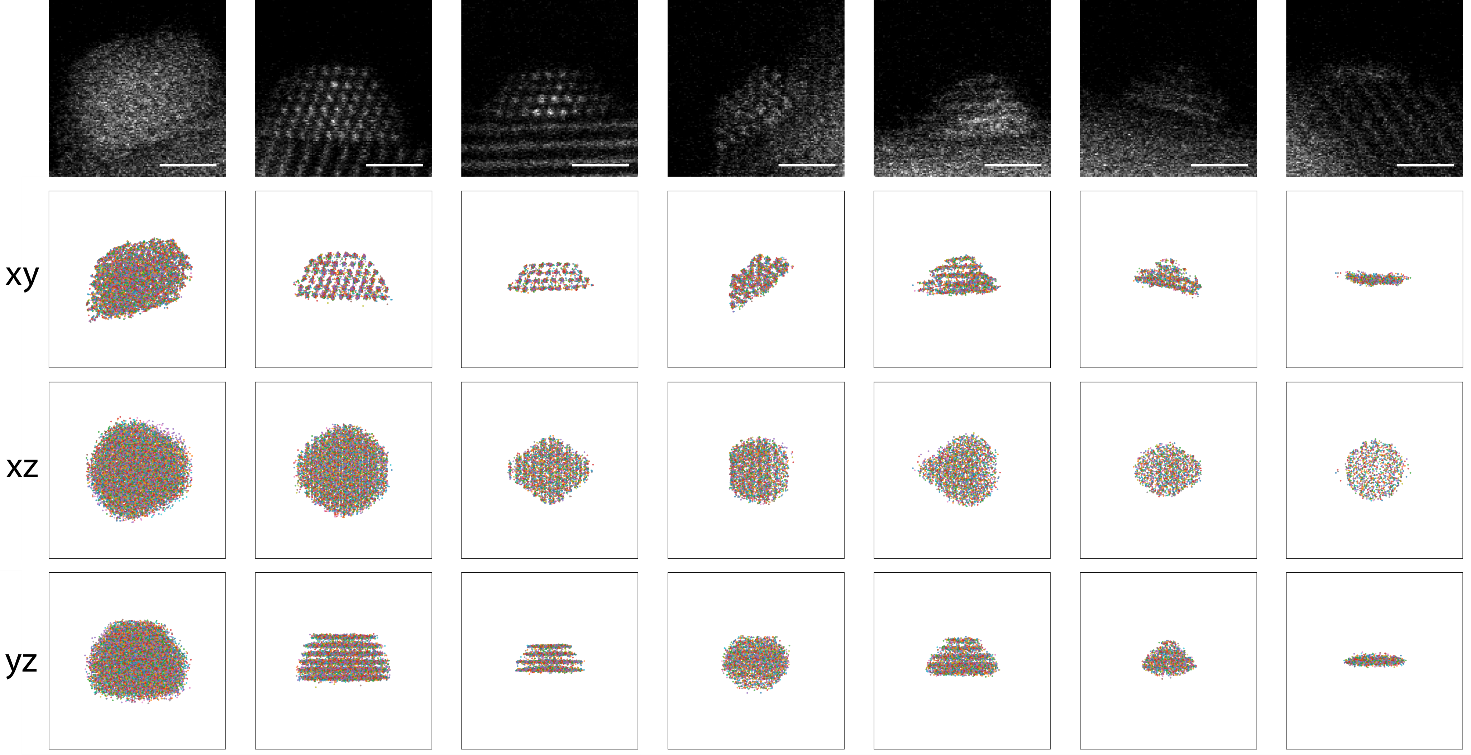


**Figure S13:** 25 overlaid structure predictions for all 7 experimentally observed images presented in figure 4.

**Video S1:** Visualization of the denoising process, bringing gaussian noise into a predicted structure over 100 time steps.

**Video S2:** Visualization of the 3D dynamics discussed in Figure 5 of the main manuscript.
